# Fetal Heart Rate Preprocessing Techniques: A Scoping Review

**DOI:** 10.3390/bioengineering11040368

**Published:** 2024-04-11

**Authors:** Inês Campos, Hernâni Gonçalves, João Bernardes, Luísa Castro

**Affiliations:** 1 Faculty of Engineering, University of Porto, 4200-465 Porto, Portugal; 2 Institute of Biomedical Sciences Abel Salazar, University of Porto, 4050-313 Porto, Portugal; 3 Center for Health Technology and Services Research (CINTESIS@RISE), Faculty of Medicine, University of Porto, 4200-319 Porto, Portugal; hernanigoncalves@med.up.pt (H.G.); jbernardes59@gmail.com (J.B.); 4 Department of Community Medicine, Information and Health Decision Sciences (MEDCIDS), Faculty of Medicine, University of Porto, 4200-319 Porto, Portugal; 5 Department of Obstetrics and Gynecology, Faculty of Medicine, University of Porto, 4200-319 Porto, Portugal; 6 Department of Obstetrics and Gynecology, São João Hospital, 4200-319 Porto, Portugal

**Keywords:** fetal heart rate, cardiotocography, preprocessing, maternal–fetal ambiguity, missing samples, artifacts

## Abstract

Monitoring fetal heart rate (FHR) through cardiotocography is crucial for the early diagnosis of fetal distress situations, necessitating prompt obstetrical intervention. However, FHR signals are often marred by various contaminants, making preprocessing techniques essential for accurate analysis. This scoping review, following PRISMA-ScR guidelines, describes the preprocessing methods in original research articles on human FHR (or beat-to-beat intervals) signal preprocessing from PubMed and Web of Science, published from their inception up to May 2021. From the 322 unique articles identified, 54 were included, from which prevalent preprocessing approaches were identified, primarily focusing on the detection and correction of poor signal quality events. Detection usually entailed analyzing deviations from neighboring samples, whereas correction often relied on interpolation techniques. It was also noted that there is a lack of consensus regarding the definition of missing samples, outliers, and artifacts. Trends indicate a surge in research interest in the decade 2011–2021. This review underscores the need for standardizing FHR signal preprocessing techniques to enhance diagnostic accuracy. Future work should focus on applying and evaluating these methods across FHR databases aiming to assess their effectiveness and propose improvements.

## 1. Introduction

Fetal heart rate (FHR) monitoring is of utmost importance for fetal well-being assessment, during pregnancy and labor. FHR analysis allows the early diagnosis of fetal distress situations, such as fetal acidosis, dystocia or preterm birth, and, consequently, prompt and adequate obstetrical intervention. In order to perform an early diagnosis of such conditions, intrapartum fetal monitoring with cardiotocography (CTG) has been widely employed. In CTG, in addition to the FHR, used to examine variability, decelerations and accelerations, uterine contractions are also commonly recorded [1].

### 1.1. Methods for FHR Acquisition

Various methods are available for monitoring FHR, which can be broadly classified into invasive and non-invasive techniques. These include auscultation methods like Doppler ultrasound and the fetoscope, as well as electronic fetal monitoring. Electronic fetal monitoring can be further subdivided into external methods, such as Doppler ultrasound and tocodynamometers, and internal methods, which involve direct fetal electrodes and intrauterine pressure catheters [2,3].

#### 1.1.1. External Ultrasound Doppler

In clinical practice, the most common and easy method of acquiring FHR signals is the well-known external ultrasound Doppler probe. The device operates on the Doppler effect principle: as the probe is placed on the maternal abdomen, ultrasound waves are emitted towards the fetus and reflected to the probe. The movement of the fetal heart is detected and the change in frequency between the emitted and the received waves is processed by the probe, creating an audible representation of the fetal heartbeat and producing an estimate of the fetal heart rate. Those signals can be heard by health professionals or printed in readouts to be analyzed [2,4].

#### 1.1.2. Transabdominal Fetal Electrocardiogram

Another promising non-invasive technique to monitor the FHR is the transabdominal fetal electrocardiogram (TA-fECG). It involves placing electrodes on the mother’s abdomen to detect the electrical activity of the fetal heart. The main challenge in TA-fECG is distinguishing the fetal heart signal from the mother’s heart signal and other noise. Advanced signal processing techniques are used to isolate and amplify the fetal signal. Once isolated, the fetal heart rate is calculated and analyzed to assess fetal well-being. This technique provides valuable information, especially in situations where ultrasound methods might have limitations. Nevertheless, the signal can still be considerably contaminated by the maternal heart rate (MHR) [3,5].

#### 1.1.3. Fetal Phonocardiography

Fetal phonocardiography (fPCG) offers a cost-effective and non-invasive approach to continuous FHR monitoring, showing great promise. The fetal heart sounds are recorded using probes placed on the surface of the mother’s body that can detect the mechanical vibrations induced by the fetal heart. This modern auscultation technique provides additional diagnostic information on congenital heart diseases. However, fPCG signal quality can be affected by several sources, including maternal motion, respiratory activity, or uterine contraction signals [6].

#### 1.1.4. Fetal Electrocardiogram

An internal and invasive, though more accurate, method of acquiring this signal consists of placing electrodes on the fetus’ scalp and obtaining a fetal electrocardiogram (fECG) [7]. This allows a more accurate identification of the RR-intervals (time between consecutive R waves of the fetal electrocardiogram QRS complex), enabling enhanced accuracy in the FHR signal obtention. However, it can only be used during labor after the rupture of the fetal membranes and implies extra costs, since it requires a disposable electrode and, by being invasive, is associated with the risk of infection. It is generally used when additional detailed monitoring of the fetal heart is necessary [8,9].

#### 1.1.5. Fetal Electrocardiogram with STAN

ST analysis (STAN) in fetal monitoring is a technique that analyzes the ST segment of the fECG during labor. This segment of the fECG waveform can indicate how well the fetal heart is tolerating the stress of labor. Changes in the ST segment, particularly to certain heart rate patterns, can suggest fetal distress related to oxygen deprivation. The goal of STAN is to improve the detection of fetuses at risk for hypoxia, potentially reducing the need for interventions like cesarean sections by providing more precise information about the fetal condition [10,11]. Despite mixed evidence regarding its effectiveness, with some studies questioning its use [12], STAN analysis through fECG continues to be supported and employed primarily in Europe for enhanced fetal monitoring during labor.

### 1.2. FHR Signal Contamination

However, despite the benefits of studying CTG records for preventing adverse perinatal outcomes, its advantages have been below initial expectations, given the complexity of the FHR signal and its contamination by several sources, such as other physiological signals, as occurs with maternal–fetal ambiguity (i.e., misinterpretation of the MHR as FHR) or artifacts [1]. Such contaminations include artifacts that could be caused by mother/fetal movement, displacement of the ultrasound probe, or simply by misdetection of the fetal heartbeat by the recording device [13].

#### 1.2.1. Outliers/Artifacts

Among these signal types of contaminations, a particular challenge is the presence of spiky artifacts—also known as outliers/artifacts, as designated in this scoping review. These are samples that significantly differ from their neighbors to an abnormal certain extent, a phenomenon whose definition and impact may vary according to different authors in the field. However, the consensual guidelines for FHR interpretation, such as those proposed by the International Federation of Gynecology and Obstetrics (FIGO) [1], consider that the removal of spiky artifacts should be performed whenever a difference between adjacent beats (or samples) exceeding 25 beats per minute (bpm) is detected. In such cases, a linear interpolation is commonly executed between the first point and the start of the next stable FHR segment (a group of five adjacent samples that differ less than 10 bpm from each other) [14].

#### 1.2.2. Missing Samples

One common characteristic of FHR recordings acquired externally through Doppler ultrasound is the frequent occurrence of missing samples, which correspond to points where the cardiotocograph was not able to detect the FHR, usually due to challenging acquisition conditions (e.g., fetal or maternal movements or sensors’ displacement) or misfunction [15]. These gaps in the recordings make it difficult to establish a continuous and accurate assessment of the FHR pattern, affecting the values of features computed such as short- and long-time variability and spectral indices [16,17,18]. Besides the higher uncertainty of the analysis when more missing values are present, there is also the risk of missing critical events such as important variations or decelerations in the heart rate, affecting the correct assessment of fetal well-being. Typically, most missing segments are removed and/or replaced by a linear interpolation between the valid samples [14,19,20].

#### 1.2.3. Maternal–Fetal Ambiguities

Maternal–fetal ambiguities present a significant challenge in FHR monitoring. This issue arises when the FHR recordings are affected by the temporary acquisition of the MHR, a situation particularly common when using external monitoring with Doppler ultrasound. Accidental capture of the MHR during external monitoring has been reported in as many as 90% of recordings taken during labor [21]. Such occurrences can lead to substantial errors in interpreting the FHR, potentially resulting in misdiagnoses ranging from newborn acidemia to fetal death [22,23,24]. These FHR-MHR ambiguities are usually detected by subtracting the MHR signals from the FHR counterparts and verifying whether the absolute difference falls within a certain threshold [5].

#### 1.2.4. Other Signal Interference

Finally, the FHR signal can be affected by some interfering signals (physiological and/or external) that lie in the same frequency range, which are difficult to remove through traditional filtering techniques (low-pass filters, for example). The application of the wavelet transform has proven to be a more flexible and effective method in the denoising of the FHR signal when compared to conventional filtering [25].

### 1.3. FHR Monitoring

**Figure 1:**
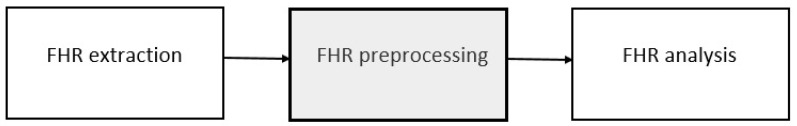
Main stages of fetal heart rate (FHR) monitoring: (1) FHR extraction; (2) FHR preprocessing after FHR has been obtained; and (3) FHR analysis.

FHR monitoring comprises three main stages: (1) FHR extraction; (2) FHR preprocessing after FHR has been obtained; and (3) FHR analysis (Figure 1). Preprocessing techniques for FHR in central monitoring systems are critical in clinical settings. These systems must generate real-time alerts, enabling healthcare professionals to act promptly to signs of fetal distress or hypoxia [26]. However, for real-time alerts to be reliable, the computational analysis must be rapid, as unreliable signals can compromise their accuracy. Moreover, evidence suggests that different sampling frequencies of the signal can significantly impact the quantification of both linear (time and frequency domain) and nonlinear indices [27]. Currently, several systems for the central monitoring of fetal signals are available, with different approaches when it comes to signal preprocessing. The first commercialized computational system was developed for antepartum monitoring, given the reduced challenges for signal preprocessing in this gestational period compared to the intrapartum period [26]. One of the pioneers in this topic was the Sonicaid System 8000, a computerized system for antenatal FHR analysis, through the analysis of RR-interval series, issued in 1991 [28]. This system was initially commercialized by Oxford Sonicaid Ltd., Abingdon, UK, and is now commercialized by the Huntleigh Healthcare company, Cardiff, UK, being widely disseminated in clinical practice in the antepartum period. The performance of this system has been assessed in several studies [29], including two randomized controlled trials [30,31] and two meta-analyses [32,33]. Its performance ensures good online clinical interactions and good quality recordings, whilst minimizing the time required to obtain the necessary information (based on fetal movements and tocodynamometer readings, as well as FHR) [28]. In fact, due to the performance of the computers at the time, the Sonicaid System 8000 preprocessing algorithm produced a shorter signal series by averaging the original signal over 3.75 s periods (rather than 2 or 4 Hz). This preprocessing phase, to reduce the signal series size for faster computation, also allows a cleaning of the signal before its processing. With modern computers with much higher processing performances, the reduction in signal series is no longer a major issue regarding the necessary processing speed for online interaction in clinical practice. Several other systems have been developed since then, both for the computer analysis of FHR in the ante- and intrapartum periods [26], namely, the ARGUS, the Guardian^TM^ and Infant^®^, the MILOU^®^, the MOSOS^®^ CTG, the OB TraceVue^®^, the OBIX^®^ Perinatal Data System, the PeriCALM^TM^, the Trium CTG Online^®^, and the Omniview-SisPorto [34,35].

### 1.4. Purpose and Study Contributions

The preprocessing stages, essential for accurate signal analysis, are significantly influenced by a range of factors. These include the method for FHR acquisition, the nature of the sampling technique (whether regular or irregular), and the types of poor signal quality events. Additionally, the fetus’ sex and the conditions under which the signal was acquired—such as intrapartum or antepartum, the gestational age, and the use of epidural anesthesia during labor—are also crucial considerations [36].

The proper detection and correction of the artifacts are essential to reconstruct the FHR signal before the computation and analysis of FHR parameters, aiming at providing a reliable fetal health status assessment and diagnosis [37]. In other words, FHR preprocessing plays a key role not only in the detection and correction of poor signal quality events, but also in the overall analysis of fetal well-being. Some literature reviews have been published focusing on FHR analysis and processing [38,39], on fECG extraction [40], providing an overview of current FHR and UC monitoring technologies (with a succinct not systematic description of preprocessing techniques [41]) or reviewing feature extraction techniques, classification and preprocessing [42]. However, to the authors’ knowledge, there is still a lack of a review identifying all of the reported methods and techniques used in the preprocessing stage of the signal concerning the detection and/or correction of missing samples, artifact-generated samples, and interference with other signals.

Therefore, the main goal of this work was to perform a scoping review of the existing FHR preprocessing techniques reported in the available literature. The focus of this review was not to address the feasibility, appropriateness, or effectiveness of a certain method or approach; instead, the goal was to identify, map, and describe the techniques reported in the literature. This allowed the in-depth study of the currently available FHR preprocessing techniques, according to the signal considered (FHR only, RR-interval, or simultaneous FHR and MHR), type of preprocessing methods reported (detection of poor signal quality events, correction of such, both detection and correction, resampling or detrending), category of poor signal quality episodes (outliers, missing samples, maternal–fetal ambiguities, or interferences with other signals), chosen sampling rate, and acquisition method.

## 2. Materials and Methods

To thoroughly identify and describe the FHR signal processing techniques that have been actively employed and reported in the field, a scoping review was conducted. This review encompassed all original research articles on human FHR (or RR-intervals) signal preprocessing found in PubMed and Web of Science, covering publications from their inception up to May 2021. Three reviewers carried out the analysis.

**Figure 2:**
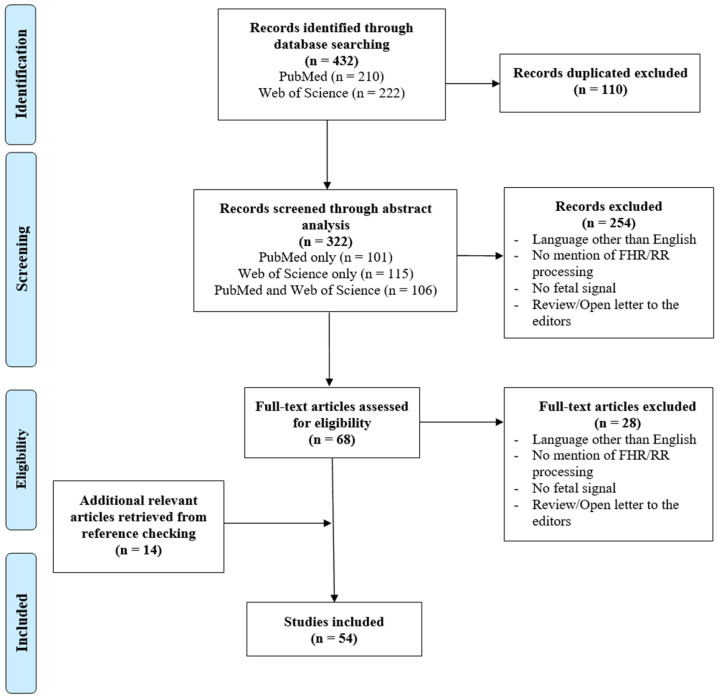
PRISMA flow diagram of the articles’ selection process in the scoping review.

The initially proposed query ((“fetal heart rate” OR “foetal heart rate” OR (fetal AND “heart rate”)) AND (preprocessing OR pre-processing)) was generalized and adapted according to the obtained results, since most did not refer to FHR preprocessing techniques. Thus, to collect the maximum number of articles related to FHR signal preprocessing, the following final query was selected: ((fetal OR foetal) AND (“heart rate” OR cardiotocography)) AND (denoising OR “noise removal” OR artifact OR ambiguities OR missing OR preprocessing OR pre-processing). As a result, 432 records, available in the online databases, were gathered, 210 of which were obtained through PubMed and 222 via Web of Science. After removing 110 duplicates, a total of 322 articles were selected for screening (101 via PubMed only, 115 via Web of Science only, and 106 from both online databases, as schematized in Figure 2).

The information from each article was organized in a tabular format, containing its title, the authors’ names, the publication year, and the respective DOI. The articles’ abstracts were then distributed between three reviewers, so that every article was independently rated by two of them. This ensured that the decision to include one article, based on its abstract, was performed based on the consensus from two reviewers, thus being more reliable. Each reviewer had the task of evaluating the abstract of an assigned article individually, determining and reporting the presence of any exclusion criteria. Subsequently, these individual assessments were deliberated in a meeting involving all three reviewers to collectively decide whether to include or exclude the article in the screening stage. The exclusion criteria for the screening phase were as follows: language other than English; reported analysis does not mention FHR/RR-interval signal preprocessing; reported analysis does not involve fetal signal; reported analysis is a review or open letter to the editors. This resulted in the exclusion of 254 articles and, consequently, a total of 68 were assessed for eligibility.

During the eligibility phase, where full-text articles were analyzed, the information was once again organized in a tabular format. This table included more specific characteristics in addition to those mentioned in the screening file. This time, the reviewers specified the type of signal referred to in the article (simply FHR, RR-intervals, or simultaneous FHR and MHR), whether it mentioned artifact detection, correction, or both, the reported signal’s sampling frequency, the resampling technique, the new/final sampling frequency, the acquisition mode and, finally, the filtering/detrending method. Moreover, the reviewers detailed the type of artifacts detected and corrected (missing samples, outliers, MHR-FHR ambiguities, and interferences with other signals). This allowed a more in-depth analysis of the gathered articles, presented in the discussion part of this study. In this phase, each of the 68 articles was read by the three reviewers, who met regularly, to decide whether or not to include an article. As a result, in the eligibility phase, 28 records were excluded (by the same criteria already presented for the screening step) and 14 were added, obtained from reference checking since the reviewers considered them to be relevant for this study and the query did not capture any of them. Finally, a total of 54 articles were included, as illustrated in Figure 2. The protocol for this review was not documented nor prospectively registered.

## 3. Results

**Figure 3:**
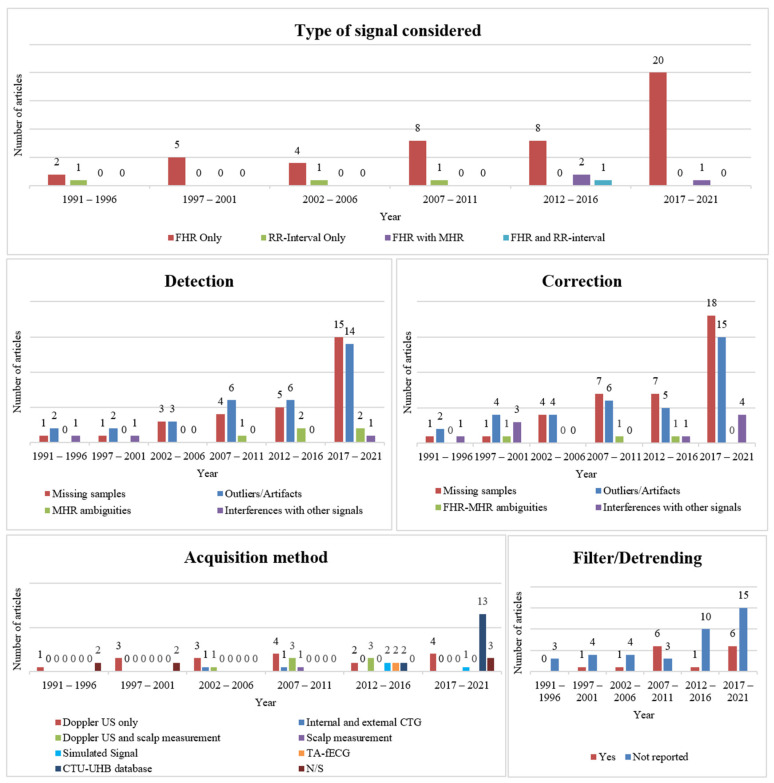
Graphical evolution of the number of articles reporting FHR preprocessing techniques throughout time, according to the type of signal considered (**top**), detection (**middle**, **left**) and correction (**middle**, **right**) techniques employed, acquisition method (**bottom**, **left**), and filters applied (**bottom**, **right**). Note that some articles addressed more than one method. Abbreviations: FHR—fetal heart rate; MHR—maternal heart rate; N/S—not specified; US—ultrasound; CTG—cardiotocography; TA-fECG—transabdominal fetal electrocardiogram.

From the 54 articles included, concerning the type of signal considered, the vast majority (*n* = 47) reported preprocessing techniques for the FHR signal. Four studies focused on RR-intervals or beat-to-beat signal analysis, whilst three articles focused on the combination of FHR and MHR. The evolution in the number of included articles regarding the type of signal considered over 5-year periods is illustrated in the top graphical representation of Figure 3. A substantial increase in the study of preprocessing techniques of only the FHR signal throughout the years is denoted. Regarding the preprocessing techniques, relative to poor signal quality events, eight articles only mentioned the correction of such, whilst three merely referred to their detection. Nevertheless, a substantially larger number of studies (*n* = 39) performed both the detection and correction of these incidents. The remaining four articles (of all those included) addressed either FHR preprocessing only through the application of analog/digital filters (*n* = 2), only using resampling techniques (*n* = 1), or referred to the signal’s representation through a shift-invariant dictionary (*n* = 1). The latter corresponds to the lines in Table 1 that do not contain any information regarding the detection and correction of poor signal quality episodes. Nonetheless, detrending using filters or resampling methods was also applied in some studies, besides episode correction/detection. Indeed, in total, 16 articles performed resampling techniques, although these were not described in some cases (*n* = 3). Likewise, a total of 15 articles mentioned the application of analog/digital filters.

Regarding the detection of poor signal quality events, there was a diverse focus among the articles: 29 of them focused on identifying missing samples, 33 addressed recognizing outliers or artifacts, 5 explored finding FHR-MHR ambiguities, and 3 examined detecting interferences with other signals. It should be noted that some articles reported on more than one of these approaches (refer to Table 1 for more details). According to the articles analyzed, these types of episodes are mainly detected when they deviate to an abnormal extent (that may vary from article to article) from the previously acquired values. The progression of the number of included articles regarding the detection of signal quality issues throughout time is presented in the middle-left graphical representation of Figure 3. The plot illustrates the growing focus on missing samples and outliers or artifact detection techniques.

**Figure 4:**
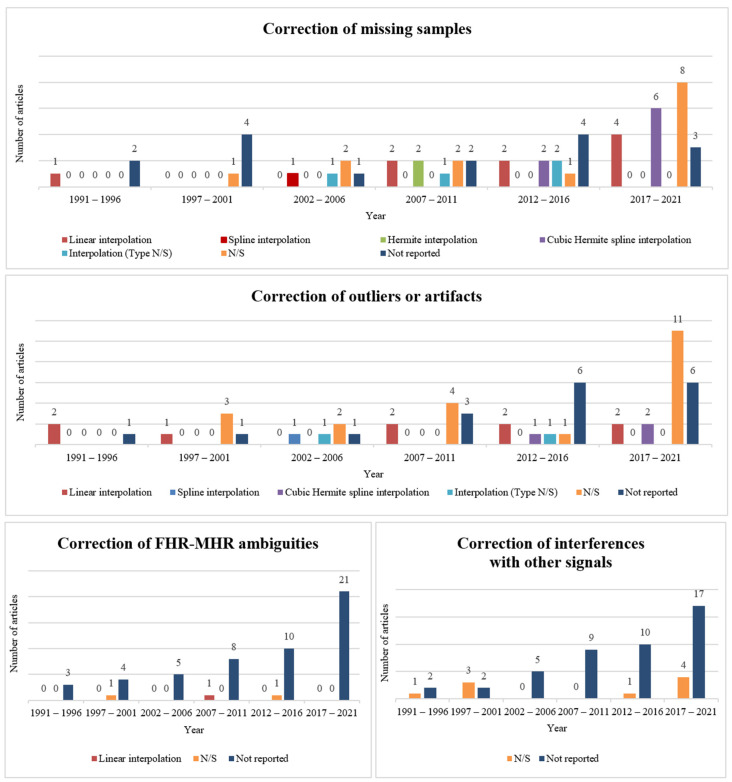
Graphical evolution of the number of articles reporting the correction method of missing samples (**top**), outliers/artifacts (**middle**), FHR-MHR ambiguities (**bottom**, **left**), and interferences with other signals (**bottom**, **right**). Abbreviations: N/S—not specified.

On the other hand, regarding the correction of such events, 38 articles studied the correction of missing samples, 36 the correction of outliers or artifacts, 3 the adjustment of FHR-MHR ambiguities and 9 the attenuation of interferences with other signals (with some of the articles addressing more than one of those topics). Moreover, it was clear that the most common method for correcting these episodes was interpolation, with a total of 30 articles reporting it. From those, 13 articles mentioned the use of linear interpolation, 8 mentioned cubic Hermite spline, 3 mentioned the use of Hermite, 2 referred to spline, and 4 did not specify the type of interpolation employed during the correction step. Figure 4 depicts the progression of the number of included studies mentioning the different types of poor signal quality event correction throughout time. It is worth highlighting that the definition of missing sample, outlier, artifact, ambiguity, and interference differs between the included articles. Therefore, in some dubious cases, it was considered that the FHR correction/detection algorithm proposed in a given article tackled more than one type of event. The evolution in the number of included articles regarding the type of corrected events throughout time is presented in the middle-right graphical representation of Figure 3. Similar to the plot for detected poor signal quality events, the graphic illustrates the growing interest over time in techniques for correcting missing samples and outliers or artifacts.

When it comes to the method of acquiring the signal specified in 47 articles, only 3 referred to the analysis of simulated signals. A considerable number of articles (*n* = 15) mentioned the study of FHR preprocessing methods using the Czech Technical University (CTU) in Prague and the University Hospital in Brno (UHB) database, known as the CTU-UHB Intrapartum Cardiotocography dataset [43]. It contains 552 cardiotocographic (CTG) recordings that start no more than 90 min before delivery, each being, at most, 90 min long. In addition, each CTG contains an FHR time series and a uterine contraction signal, each sampled at 4 Hz. The database is composed of a mixture of recordings acquired by a Doppler Ultrasound probe, a direct scalp measurement, or a combination of both. The remaining articles acquired the signal externally (*n* = 18), mainly through a Doppler ultrasound probe, or both externally and internally using scalp electrodes (*n* = 11). The evolution in the number of included articles regarding the type of acquisition method throughout time is illustrated in the bottom-left graphical representation of Figure 3.

Additionally, most studies (*n* = 39) did not refer to the use of filtering or detrending techniques for signal preprocessing, as presented in the bottom-right graphical representation of Figure 3.

Finally, regarding the used signals, the majority of studies (*n* = 29) referred to the original sampling frequency of the signal as being 4 Hz. The full characteristics of the included studies are presented in Table 1. The table contains all of the main characteristics retrieved by the reviewers for each included study.

**Table 1:** Study characteristics of the 54 included articles.

Year, Authors (Ref)	Type of Signal Considered	Detection			Correction			Filtering/Detrending	Acquisition Method or Selected Dataset
		Missing Samples	Outliers/ Artifacts	MHR Ambiguities| Interferences with Other Signals	Missing Samples	Outliers/ Artifacts	MHR Ambiguities| Interferences with Other Signals		
Boudet et al. 2020 [44]	FHR only	-	Yes, N/S	Yes, N/S|-	Linear interpolation	Yes, N/S	-	-	-
Guijarro-Berdiñas et al. 1997 [37]	FHR only	-	Yes, N/S	-|-	Yes, N/S	Yes, N/S	Yes, N/S|-	-	Doppler ultrasound
Cömert et al. 2017 [45]	FHR only	Yes, N/S	Yes, N/S	-|-	Cubic Hermite spline interpolation	Cubic Hermite spline interpolation	-|Yes, N/S	-	CTU-UHB database
Cömert et al. 2019 [46]	FHR only	Yes, N/S	Yes, N/S	-|-	Cubic Hermite spline interpolation	Yes, N/S	-|-	-	CTU-UHB database
Spilka et al. 2009 [47]	FHR only	-	Yes, N/S	-|-	Hermite interpolation	Yes, N/S	-|-	Third order polynomial	Doppler ultrasound and scalp measurement
Agostinelli et al. 2017 [48]	FHR only	-	-	-|-	Linear interpolation	Yes, N/S	-|-	-	CTU-UHB database
Frigo et al. 2017 [49]	FHR only	-	-	-|-	Yes, N/S	-	-|-	Yes, N/S	CTU-UHB database
Marques et al. 2019 [50]	FHR only	Yes, N/S	Yes, N/S	-|-	Linear interpolation	Linear interpolation	-|-	Low pass filter and Hilbert Transform	Doppler ultrasound
Cesarelli et al. 2007 [51]	FHR only	-	Yes, N/S	-|-	Linear interpolation	Linear interpolation	-|-	Fifth-order median filter	Doppler ultrasound
Moczko et al. 2002 [52]	FHR only	-	-	-|-	-	-	-|-	Digital bidirectional autoregressive first-order filter	Doppler ultrasound
Lu et al. 2020 [53]	FHR only	Yes, N/S	-	-|-	Cubic spline interpolation	-	-|Yes, N/S	-	CTU-UHB database
Wrobel et al. 2015 [54]	FHR only	-	-	-|-	Linear interpolation	-	-|-	-	CTU-UHB database
Chudáček et al. 2009 [55]	FHR only	Yes, N/S	Yes, N/S	-|-	Hermite interpolation	Yes, N/S	-|-	Third-order polynomial	Doppler ultrasound and scalp measurement
Papadimitriou et al. 1996 [25]	FHR only	-	-	-|Yes, N/S	-	-	-|Yes, N/S	-	Doppler ultrasound
Nokas et al. 2002 [56]	FHR only	Yes, N/S	Yes, N/S	-|-	Yes	Yes, N/S	-|-	-	Doppler ultrasound
Cömert et al. 2019 [57]	FHR only	Yes, N/S	Yes, N/S	-|-	Cubic Hermite spline interpolation	Yes, N/S	-|-	Median filter	CTU-UHB database
Fergus et al. 2018 [58]	FHR only	-	-	-|-	Cubic Hermite spline interpolation	Cubic Hermite spline interpolation	-|-	Finite Impulse Response sixth-order high-pass filter	CTU-UHB database
Cömert et al. 2018 [59]	FHR only	Yes, N/S	Yes, N/S	-|-	Cubic Hermite spline interpolation	Yes, N/S	-|Yes, N/S	Median filter	CTU-UHB database
Feng et al. 2017 [60]	FHR only	-	-	-|-	Yes	-	-|-	-	CTU-UHB database
Spilka et al. 2012 [18]	FHR only	-	Yes, N/S	-|-	Linear interpolation	Yes, N/S	-|-	-	CTU-UHB database
Tan et al. 2021 [61]	FHR only	Yes, N/S	Yes, N/S	-|-	-	-	-|-	-	-
Feng et al. 2021 [15]	FHR only	Yes, N/S	-	-|-	Yes, N/S	-	-|-	-	CTU-UHB database
Zhao et al. 2019 [62]	FHR only	Yes, N/S	Yes, N/S	-|-	Yes, N/S	Yes, N/S	-|-	-	CTU-UHB database
Tang et al. 2018 [63]	FHR only	Yes, N/S	Yes, N/S	-|-	Yes, N/S	Yes, N/S	-|-	Savitzky–Golay filter	Doppler ultrasound
Georgoulas et al. 2017 [64]	FHR only	Yes, N/S	Yes, N/S	-|-	Yes, N/S	Yes, N/S	-|-	-	CTU-UHB database
Krupa et al. 2009 [65]	FHR only	Yes, N/S	-	-|-	Yes, N/S	-	-|-	Butterworth low-pass filtering	Doppler ultrasound
Jezewski et al. 2008 [66]	FHR only	-	Yes, N/S	-|-	-	Yes, N/S	-|-	-	Doppler ultrasound
Papadimitriou et al. 1999 [67]	FHR only	-	-	-|-	-	Yes, N/S	-|Yes, N/S	Low-pass filter	-
Papadimitriou et al. 1997 [68]	FHR only	-	-	-|-	-	Yes, N/S	-|Yes, N/S	-	Doppler ultrasound
Ayres-de-Campos et al. 2017 [35]	FHR only	Yes, N/S	Yes, N/S	-|-	-	Yes, N/S	-|-	-	-
Agostinelli et al. 2017 [48]	FHR only	Yes, N/S	Yes, N/S	-|-	Linear interpolation	Linear interpolation	-|-	-	CTU-UHB database
Warrick et al. 2011 [69]	FHR only	-	-	-|-	-	-	-|-	Low-order Chebyshev polynomial	Internal and external CTG
Papadimitriou et al. 1997 [70]	FHR only	-	-	-|Yes, N/S	-	-	-|Yes, N/S	-	Doppler ultrasound
Spilka et al. 2012 [71]	FHR only	-	Yes, N/S	-|-	Cubic Hermite spline interpolation	Linear interpolation	-|-	Second-order polynomial	Doppler ultrasound and scalp electrode
Bernardes et al. 1991 [34]	FHR only	-	Yes, N/S	-|-	-	Linear interpolation	-|-	-	-
Romano et al. 2016 [72]	FHR only	Yes, N/S	Yes, N/S	-|-	Interpolation	-	-|-	-	Doppler ultrasound
Ayres-de-Campos et al. 2000 [14]	FHR only	Yes, N/S	Yes, N/S	-|-	-	Linear interpolation	-|-	-	-
Romano et al. 2013 [19]	FHR only	Yes, N/S	Yes, N/S	-|-	Interpolation	Interpolation	-|-	-	Simulated signal
Spilka et al. 2013 [20]	FHR only	Yes, N/S	Yes, N/S	-|-	Cubic Hermite spline interpolation	Cubic Hermite spline interpolation	-|-	-	Doppler ultrasound and scalp electrode
Urdal et al. 2019 [73]	FHR only	Yes, N/S	Yes, N/S	-|-	Yes, N/S	Yes, N/S	-|-	-	Doppler ultrasound
Oikonomou et al. 2013 [74]	FHR only	Yes, N/S	-	-|-	Yes, N/S	-	-|-	-	Simulated signal
Gonçalves et al. 2006 [7]	FHR only	Yes, N/S	Yes, N/S	-|-	Spline interpolation	Spline interpolation	-|-	-	Doppler ultrasound and scalp electrode
Nunes et al. 2014 [75]	FHR only	Yes, N/S	-	-|-	-	-	-|-	-	Doppler ultrasound and scalp electrode
Warrick et al. 2009 [76]	FHR only	Yes, N/S	Yes, N/S	Yes, N/S |-	Linear interpolation	Linear interpolation	Linear interpolation |-	High- and low-pass filters	Doppler ultrasound
Urdal et al. 2021 [77]	FHR only	-	-	-|-	-	-	-|-	-	Simulated signal
Cesarelli et al. 2007 [78]	FHR only	Yes, N/S	Yes, N/S	-|-	Yes, N/S	Yes, N/S	-|-	-	Doppler ultrasound and scalp electrode
Cao et al. 2003 [79]	FHR only	-	-	-|-	Yes, N/S	Yes, N/S	-|-	-	Doppler ultrasound
Felgueiras et al. 1996 [80]	RR-interval only	Yes, N/S	Yes, N/S	-|-	Linear interpolation	Linear interpolation	-|-	-	-
Peters et al. 2004 [81]	RR-interval only	Yes, N/S	Yes, N/S	-|-	Interpolation	Interpolation	-|-	-	Internal and external CTG
Peters et al. 2011 [82]	RR-interval only	-	-	-|-	Interpolation	-	-|-	-	Scalp measurement
Casati et al. 2014 [83]	RR-interval and FHR	-	Yes, N/S	-|-	-	Linear interpolation	-|Yes, N/S	-	TA-fECG
Reinhard et al. 2013 [21]	FHR with MHR	-	-	Yes, N/S |-	-	-	-|-	-	External measurements TA-fECG
Pinto et al. 2015 [5]	FHR with MHR	-	-	Yes, N/S |-	-	-	Yes, N/S |-	-	Doppler ultrasound
Barzideh et al. 2018 [17]	FHR with MHR	Yes, N/S	Yes, N/S	Yes, N/S|Yes, N/S	Yes, N/S	Yes, N/S	-|Yes, N/S	-	Doppler ultrasound

Abbreviations: FHR—fetal heart rate; N/S—not specified; CTG—cardiotocography; TA-fECG—transabdominal fetal electrocardiogram; CTU—Czech Technical University; UHB—University Hospital in Brno; MHR—Maternal heart rate.

## 4. Discussion

A total of 54 articles were included in this scoping review, referring to different methods of preprocessing the FHR signal. According to the results obtained, the FHR signal stands out as the most commonly used for studying such techniques, as reported in almost 90% of the analyzed articles, when compared to the RR-interval signal or the FHR with MHR. Moreover, two articles mentioned the preprocessing of the RR-interval signal [80,82], whilst two focused on computing the FHR signal first and then preprocessing it [81]. Additionally, three out of these four articles refer to the resampling methods that were applied to the RR-interval signal to obtain the FHR signal.

Besides this, the number of articles that focused on the correction of low signal quality events, rather than on their detection, is larger, although these are closely related. This may be because there is more space for innovation regarding correction methods compared to detection ones, which are more standardized. Indeed, the large majority of detection techniques involve the classification of a certain point as a missing sample or outlier/artifact when it is not recorded or deviates to an abnormal extent from its neighbor sample. Nonetheless, the large majority of articles (around 70%) refer to the study of both the detection and the following correction of the poor signal quality episodes.

Few articles referred solely to the application of analog/digital filters or resampling techniques for FHR preprocessing (about 5%). However, the percentage of articles that mentioned filtering and/or resampling methods before the detection/correction techniques was much higher (57%). Regarding the detection of poor signal quality events, around 60% of studies involved the discovery of missing samples and 50% that of outliers/artifacts, with only 10% detecting MHR ambiguities and 6% interferences with other signals, perhaps due to the higher popularity of the former. Likewise, most studies focused on the correction of missing samples (approximately 70%) and outliers or artifacts (65%). Although some of the correction techniques suggested were quite complex, more than half of the analyzed articles (55%) simply performed linear, spline, or cubic Hermite spline interpolation. FHR acquisition, which was only reported in 85% of the studies, proved to be mainly achieved both externally using a Doppler ultrasound probe and internally through scalp electrodes, with the most common original signal’s sampling frequency equal to 4 Hz. This is the case for the widely used CTU-CHB Intrapartum Cardiotocography dataset, which was reported in 33% of the studies. The lack of description regarding the methods employed for the correction and/or detection of artifacts in several papers hampers a more detailed and interesting discussion.

The articles that mentioned any preprocessing of fECG (rather than FHR or RR-interval preprocessing) in the abstract were excluded [84]. The noninvasive abdominal electrocardiogram (AECG) is used to produce RR-interval data and allows the recording of MHR, since the maternal electrocardiogram (mECG) is also detected from the AECG. It can be advantageous to extract fECG from AECG, rather than using Doppler ultrasound [85], although the acquisition of the former is not that easy, and the signal presents a very low signal-to-noise ratio. The fECG is heavily contaminated by the interference caused by mECG, electromyogram, and motion artifacts, which may result in a poor FHR estimation. Nonetheless, several reports presented novel signal processing techniques to tackle such issues. Some of the most popular methods are filtering techniques, including adaptive Kalman filtering and wavelet transforms. Besides this, blind source separation, including principal component analysis and independent component analysis, has been used for fECG extraction from the AECG [85]. In other words, studies are reporting the analysis of fECG and automatic feature extraction, rather than simply FHR preprocessing.

None of the included studies reported extracting signals through phonocardiography. However, the primary reason for excluding these articles from this review was that their focus was on preprocessing to obtain FHR or RR from sound signals, rather than on the preprocessing of the FHR signal itself. The non-invasive fPCG comprises the recording of fetal heart sounds: the first sound is caused by the closure of the mitral and tricuspid (atrio-ventricular) valves, while the second sound is produced by the closure of the aortic and pulmonary (semilunar) valves [86]. Since the signal is easily contaminated by fetal and maternal movements, fetal and maternal respiration, and other sources, fPCG is typically processed before FHR extraction [87]. Decomposition wavelet-based techniques are commonly referred to in the literature to denoise fPCG as well as detect fetal heart sound peaks to compute the FHR signal [86,88,89,90,91]. Methods such as adaptive support vector regression, empirical mode decomposition, and finite impulse response filters have also reportedly been used to process the fPCG signal [86,92,93].

The main limitation of this review is the possibility that some articles might have been wrongly excluded during the screening step, given that they did not include the terms “FHR” or “preprocessing” in the abstract. Another possible limitation is the lack of available articles on this topic in the chosen online databases (PubMed and Web of Science). Indeed, 14 articles, which is roughly a quarter of all of the studies included, not retrieved by the query but found by reference checking, complied with the inclusion criteria and, thus, were included in the analysis.

Nevertheless, this review presents an in-depth analysis of the available studies regarding FHR preprocessing techniques, reflecting the recent rise in the number of publications on the topic. Almost 60% of the studied articles were published in the last 10 years, with the oldest article dating back to 1991. This is a great indicator of the growing care for monitoring the fetus’ health state and preventing fetal distress situations. Besides this, a considerable number of articles referred to follow the preprocessing algorithm proposed by Bernardes J. et al. in 1991 [34] (or similar articles by the same authors), which means there is still some space for innovation in the area. Notably, it can be assumed that FHR preprocessing techniques will keep raising interest in the research community and, in the future, it might be possible to reconstruct the signal (almost) completely or reduce the noise substantially. Some studies even suggest that AECG may soon replace the popular Doppler ultrasound method, while ensuring the accuracy of the FHR is similar to that of direct electrocardiogram [87].

The main aim of the present scoping review was to describe the used FHR preprocessing techniques without performing a meta-analysis, i.e., without an explicit evaluation and comparison of different preprocessing techniques in terms of their effectiveness. Such an evaluation could be conducted by either relying on the “self-reported” performance of each technique or implementing and testing the different methods on the same database. The first approach would not be feasible since few studies provide such metrics. The second approach would require the strenuous implementation of all of the techniques with an adequate study of the parameters’ sensitivity, based on a (preferably) open dataset with simulated and real signals specifically elaborated for such a purpose. Nevertheless, this could constitute an interesting and challenging approach for further research.

## 5. Conclusions

To conclude, in the current scoping review, a total of 54 articles reporting FHR preprocessing were included, based not only on the query’s result in PubMed and Web of Science, but also on reference checking from the latter. Several different approaches for preprocessing FHR were identified, including the detection and correction of poor signal quality events, as well as detrending and resampling methods. Among the techniques most frequently reported in the included articles, artifact detection typically involved analyzing deviations from adjacent samples, while correction was commonly achieved through linear or cubic Hermite spline interpolation methods. This suggests that a substantial range of FHR signal preprocessing techniques are available, highlighting the increasing interest in this topic within the research community. It is worth noting the lack of consensus regarding the definition of missing samples, outliers, and artifacts, which may vary according to the article’s authors. The lack of consensus regarding the definition of missing samples, outliers, and artifacts in the data, particularly in fields like signal processing or data analysis, can lead to numerous potential implications and challenges. This inconsistency can lead to disparate conclusions and conflicting results across analyses, compromising the reproducibility of studies and hampering the aggregation of results in literature reviews and meta-analyses. Overall, a consensus on these definitions is crucial for ensuring the reliability, comparability, and reproducibility of data-driven research and analysis. Further work would involve the implementation of algorithms based on these preprocessing methods on an FHR database, as well as their comparison. By doing so, one could evaluate the accuracy of the currently available FHR preprocessing approaches, whilst suggesting further improvements.

## Data Availability

The raw data supporting the conclusions of this article will be made available by the authors upon reasonable request.

## Abbreviations

AECG	Abdominal electrocardiogram
bpm	Beats per minute
CTG	Cardiotocography
CTU-UHB	Czech Technical University and University Hospital in Brno
fECG	Fetal electrocardiogram
FHR	Fetal heart rate
FIGO	International Federation of Gynecology and Obstetrics
mECG	Maternal electrocardiogram
MHR	Maternal heart rate
STAN	ST analysis
TA-fECG	Transabdominal fetal electrocardiogram
